# Supplementary material for: ZIP4 is required for normal progression of synapsis and for over 95% of crossovers in wheat meiosis
Source: Front Plant Sci. 2023 May 30;14:1189998. doi: 10.3389/fpls.2023.1189998 (PMC10266424; doi:10.3389/fpls.2023.1189998)
Supplement: Supplementary file 2 [file Table_1.docx]

**Supplementary Table 1**

Primers for detection of CRISPR mutations in the *TtZIP4-B2* gene in the primary transgenics (T_0_) and subsequent generation (T_1_) using Sanger and Illumina sequencing

|  |  | **Sequences (5’-3’)** |
| --- | --- | --- |
| Sanger Sequencing | Kronos_5BF1 | AAGCGCGCCAACTCCGCCGC |
|  | Kronos_5BR1 | CGGTGGCGAGGTCGACGCGG |
|  | Kronos_5BF2 | CCTGCTTCGACAAGGCCAC |
|  | Kronos_5BR2 | GAGGGACTTGGAGCGGCCGA |
| Illumina NGS | Kronos_5BF1 | AAGCGCGCCAACTCCGCCGC |
|  | Kronos_5BR1 | CGGTGGCGAGGTCGACGCGG |
|  | Kronos_5BF2 | CCTGCTTCGACAAGGCCAC |
|  | Kronos_5BR2 | GAGGGACTTGGAGCGGCCGA |
