# Supplementary material for: ZIP4 is required for normal progression of synapsis and for over 95% of crossovers in wheat meiosis
Source: Front Plant Sci. 2023 May 30;14:1189998. doi: 10.3389/fpls.2023.1189998 (PMC10266424; doi:10.3389/fpls.2023.1189998)
Supplement: Supplementary file 3 [file Table_2.docx]

**Supplementary Table 2**

Genome-specific primer sequences for KASP genotyping *ZIP4* tetraploid wheat lines

| **Gene** | **Chromosome location** | **KASP primer** | **Sequences (5’-3’)** |
| --- | --- | --- | --- |
| *TtZIP4-A1* | 3A | Wt primer (Vic tail) | acaattaacatgtatatttattac |
|  |  | Alt primer (FAM tail) | acaattaacatgtatatttattat |
|  |  | Common primer | tactgcttcttacgtttga |
| *TtZIP4-B1* | 3B | Wt primer (Vic tail) | gagggcgaatatccatgtgagg |
|  |  | Alt primer (FAM tail) | gagggcgaatatccatgtgaga |
|  |  | Common primer | atcttcatccattttataccaacg |
| *TtZIP4-B2*  *(ph1c*) | 5B | Wt primer (Vic tail) | gcattgctccatgtctgcta |
|  |  | Alt primer (FAM tail) | gcattgctccatgtctgctg |
|  |  | Common primer | cggcatgttgagagcatgaaga |
| *TtZIP4-B2*  *(CRISPR)* | 5B | Wt primer (Vic tail) | gcgaggttgaggccgagca |
|  |  | Alt primer (FAM tail) | gcgaggttgaggccgagc |
|  |  | Common primer | ccacggaggatgagcgacg |
