# Supplementary material for: ZIP4 is required for normal progression of synapsis and for over 95% of crossovers in wheat meiosis
Source: Front Plant Sci. 2023 May 30;14:1189998. doi: 10.3389/fpls.2023.1189998 (PMC10266424; doi:10.3389/fpls.2023.1189998)
Supplement: Supplementary file 4 [file Table_3.docx]

**Supplementary Table 3**

Genotypic effects on meiotic metaphase I chromosomes of Kronos wild type and Kr3161 *TtZIP4-A1B1B2* control plants. Mean numbers of univalents, rod and ring bivalents, trivalents and tetravalents were scored along with chiasma frequency scored as single and double chiasmata. Standard error (SE) values are shown. The range is shown in brackets. P-values < 0.05 indicate significant differences. Superscript letters a and b indicate where the significant differences lie. For scores with the same letter, the difference between the means is not statistically significant. If the scores have different letters, they are significantly different.

| **Genotype** | **No. of cells scored** | **Univalents**  Mean ± SE  (Range) | **Rod bivalents**  Mean ± SE  (Range) | **Ring bivalents**  Mean ± SE  (Range) | **Trivalents**  Mean ± SE  (Range) | **Tetravalents**  Mean ± SE  (Range) | **Single chiasmata**  Mean ± SE  (Range) | **Double chiasmata**  Mean ± SE  (Range) |
| --- | --- | --- | --- | --- | --- | --- | --- | --- |
| Kronos  wild type | 162 | 0.19 ± 0.05  (0-2) | 1.56 ± 0.10  (0-7) | 12.35 ± 0.10  (7-14) | 0.00 ± 0.00  (-) | 0.00 ± 0.00  (-) | 26.25 ± 0.11^a^  (21-28) | 28.76 ± 0.13^a^  (23-32) |
| Kr3161 control  *(ZIP4-A1B1B2)* | 168 | 0.31 ± 0.06  (0-2) | 1.74 ± 0.10  (0-6) | 12.10 ± 0.10  (8-14) | 0.00 ± 0.00  (-) | 0.00 ± 0.00  (-) | 25.94 ± 0.12^b^  (21-28) | 28.07 ± 0.12^b^  (23-31) |
| *p-value* |  | 0.1150 | 0.1491 | 0.0683 | - | - | 0.0486 | 0.0001 |
